# AAPM Medical Physics Practice Guideline MPPG 17.a: Quality management for mammography review workstation displays

**DOI:** 10.1002/acm2.14625

**Published:** 2025-03-12

**Authors:** Rebecca Milman, Nicholas B. Bevins, Mosa Alhamami, Nathan Busse, Andreea Dohatcu, Katie W. Hulme, Mary Ellen Jafari, Steven M. LaFontaine, Richard D. Nawfel, Jeffrey S. Nelson, Megan K. Russ, Michael Silosky, John M. Wait

**Affiliations:** ^1^ University of Colorado Anschutz Medical Campus Aurora Colorado USA; ^2^ Maine Medical Center Portland Maine USA; ^3^ Lahey Hospital and Medical Center Newton Massachusetts USA; ^4^ Colorado Associates in Medical Physics Denver Colorado USA; ^5^ Yale University New Haven Connecticut USA; ^6^ The Cleveland Clinic Chagrin Falls Ohio USA; ^7^ Kaiser Permanente Southern California Medical Group Pasadena California USA; ^8^ Therapy Physics Signal Hill California USA; ^9^ Brigham & Women's Hospital Boston Massachusetts USA; ^10^ Duke University Health System Durham North Carolina USA

**Keywords:** display, guideline, mammography, MPPG, MPPG 17.a, quality assurance, quality control, quality management, RWS

## Abstract

The Mammography Quality Standards Act (MQSA) sets quality standards for displays used to interpret mammography images. With the shift to digital mammography and the widespread use of remote reading workstations (RWS), updated quality management (QM) programs are needed to ensure consistent image presentation and accurate interpretation. This document recommends a QM framework for mammography RWS displays, addressing challenges such as remote environments, regulatory compliance, and evolving technology. The QM model highlights the central role of medical physicists in program design, oversight, and data review. It emphasizes periodic quality assurance (QA) and quality control (QC) procedures and training for interpreting physicians and staff. A structure for QM for remote RWS, including guidance on environmental conditions, hands‐on testing, and remote monitoring solutions, is included. The proposed program balances scientific rigor, cost‐effectiveness, and practical implementation, maintaining image quality and safety. By providing a structured approach to RWS display management, this framework supports regulators, accreditation bodies, and healthcare facilities in adapting to advancements in mammography technology while addressing logistical and operational challenges.

## 1 ABOUT MPPGS

The American Association of Physicists in Medicine (AAPM) is a nonprofit professional society whose primary purpose is to advance the science, education, and professional practice of medical physics.The AAPM has roughly 10 000 members and is the principal organization of medical physicists in the United States.

The AAPM will periodically define new practice guidelines for medical physics practice to help advance the science of medical physics and to improve the quality of service to patients throughout the United States. Existing medical physics practice guidelines will be reviewed for the purpose of revision or renewal, as appropriate, on their fifth anniversary or sooner.

Each medical physics practice guideline represents a policy statement by the AAPM, has undergone a thorough consensus process in which it has been subjected to extensive review, and requires the approval of the Professional Council. The medical physics practice guidelines recognize that the safe and effective use of diagnostic and therapeutic radiology requires specific training, skills, and techniques, as described in each document. Reproduction or modification of the published practice guidelines and technical standards by those entities not providing these services is not authorized.

The following terms are used in the AAPM practice guidelines:

1. Must and must not: Used to indicate that adherence to the recommendation is considered necessary to conform to this practice guideline. **While must is the term to be used in the guidelines, if an entity that adopts the guideline has shall as the preferred term, the AAPM considers that must and shall have the same meaning**.
2. Should and should not: Used to indicate a prudent practice to which exceptions may occasionally be made in appropriate circumstances.

## 2 INTRODUCTION

In the 1990s, the Mammography Quality Standards Act (MQSA) established new rules for healthcare facilities providing screening and diagnostic mammography services.^1^ Quality standards for personnel and equipment are an important component of MQSA. These standards were written in an era of screen‐film mammography many years before the now‐widespread use of digital mammography, picture archiving and communication system (PACS), and computer‐based review workstations (RWS). Due to the evolution of technology since the 1990s, regulators need to adapt portions of the regulations to apply to today's mammography environment. This document addresses requirements for quality management (QM) for RWS displays. Because of their role in the imaging chain, RWS displays are treated in a manner similar to film processors in the original rules. In the case of film processors, the regulations only require an MQSA‐qualified medical physicist (hereafter referred to as a medical physicist) to be physically present during a mammography equipment evaluation (MEE).^2^

Expanding the original MEE‐only requirement, many mammography units, display, and PACS vendors also require annual in‐person testing of RWS displays as part of their manufacturer‐specified quality program for digital mammography devices. Reading rooms have typically been located close to mammography areas, and the use of remote interpretation was limited, both of which reduced the burden on the medical physicist to test each RWS display. Early displays also often lacked advanced internal controls for accurate calibration and performance stability, making the physicist's tests more likely to uncover issues that would otherwise go undetected.

There have been two major changes in RWS implementation over the last several years: First, the stability of medical displays has greatly improved, including an improvement in self‐calibration through integrated photometers and remote monitoring capabilities. Second, the number of remote RWS displays nearly doubled between 2020 and mid‐2022,^3^ with many RWS now located in remote locations (e.g., radiologists’ homes). The increase in the number of RWS displays located outside of the clinical setting has created a situation where a medical physicist must travel to remote locations to perform required testing. This has introduced or magnified several challenges, including:

1. long travel times, particularly relative to the duration of testing;
2. potential shortage of medical physicists available to perform this work^4^;
3. increased regulatory burden, including credentialing requirements and costs, to support RWS displays located in a state different from that of the supported mammography equipment or used to interpret images acquired in multiple states;
4. safety and liability concerns for medical physicists working in private homes;
5. safety, liability, and privacy concerns for radiologists; and
6. working outside normal hours to accommodate radiologist schedules.

While the proliferation of remote RWS provided impetus for this report, the program described here is appropriate for all mammography RWS, regardless of their location.

A QM program includes both quality assurance (QA) and quality control (QC) activities that contribute to ensuring quality and safety and should be scientifically rigorous, easily implemented and administered, affordable, and cost‐effective.^5^ The current model is costly to the healthcare system and creates substantial and unnecessary barriers to facilities’ ability to comply with state and federal regulations without adding to the quality or safety of mammography.

The purpose of a display QM program is to ensure that mammographic images are presented in a consistent manner that maximizes the image information available to the interpreting physician. A robust program should include periodic tests that will inform the user if the quality of the display or reading conditions is compromised in a way that may affect accurate image interpretation. As image display technology changes, so do the features that are most likely to “fail” a QC test. Consequently, the tests that should be performed, their frequencies, and the appropriate oversight by a medical physicist also change. Finally, a display QM program must balance resources to achieve an acceptable outcome without undue cost or effort.

This document recommends a QM program for RWS displays used in mammography. These recommendations describe a set of QA and QC procedures that have been deemed necessary and sufficient to ensure display quality. The foundation of the QM program rests with the medical physicist,^6^ who should be responsible for establishing a facility‐specific program and overseeing the collection, analysis, and interpretation of data. Some tasks may be delegated to other appropriately trained individuals, under the general supervision of the medical physicist.^7^

AAPM AP 125‐A defines three medical physicist supervision levels for clinical procedures.^7^ Unless stated otherwise, this document assumes these definitions. It is important to note that some regulatory and accreditation agencies have different definitions for levels of supervision. For example, MQSA requires that during “direct supervision,” the supervisor “is present to observe and correct” the individual being supervised who is performing the task.^8^ AAPM defines this level of supervision as “personal supervision.” The medical physicist should ensure the levels of supervision outlined in the QM program are clearly defined and adhere to applicable state, and federal regulations.

The tests, frequencies, and oversight levels listed in this document do not supplant existing requirements. The intent is for regulators, accreditation agencies, and vendors to consider the proposed structure when creating new or revised QM programs. Similarly, the medical physicist should consider this framework when evaluating and establishing a QM program.

## 3 EXISTING TECHNICAL AND PERFORMANCE STANDARDS

This practice guideline does not intend to establish new or revised performance standards. Those creating or modifying a QM program should refer to existing technical standards or reports from groups such as AAPM when establishing performance criteria for mammography RWS displays. At the time of this practice guideline's creation, AAPM Report 270,^9^ “Display Quality Assurance,” is the most current and comprehensive AAPM performance standard encompassing medical displays. In addition, the ACR‐AAPM‐SIIM Technical Standard for Electronic Practice of Medical Imaging^10^ and the ACR‐AAPM Technical Standard for Diagnostic Interpretation Displays both serve as references for performance criteria. Overall performance capabilities of mammography display workstations are also described in the ACR‐AAPM‐SIIM Practice Parameter for Determinants of Image Quality in Mammography.^11^ These reports specify key parameters for QM of medical imaging displays, including those used for reading mammography images. References provided here^9^, ^10^, ^11^ are the most recent versions at the time of this practice guideline's writing.

## 4 REGULATORY CONSIDERATIONS SPECIFIC TO MAMMOGRAPHY DISPLAYS

Performance and technical recommendations for medical displays are not unique to mammography. However, displays used to interpret mammograms are subject to additional regulatory requirements because the U.S. Food and Drug Administration (FDA) classifies them as devices used in the acquisition, processing, or display of digital mammographic images under MQSA. As such, the FDA requires such displays to have an established QM program that is “*substantially the same as the quality assurance program recommended by the image receptor manufacturer*.^12^” Effective September 10, 2024 displays used in mammography must additionally “*have met the applicable FDA premarket authorization requirements for medical devices of that type with that intended use*” (MQSA Final Rule, 88 FR 15126, 21 CFR 900.12(b)(2)(i)). Premarket authorization requirements are outlined in 21 CFR 892.2050 and FDA's guidance “Display Devices for Diagnostic Radiology.^12^, ^13^”

The QM requirements for mammographic displays are dictated by the QM manual for the mammography unit used to generate the images. Either the required tests, frequencies, and individual responsible will be explicitly listed in the manual, or it will defer to the requirements established by the display manufacturer or vendor. For facilities using the ACR alternative standard, display QM requirements are defined by the ACR Digital Mammography QC Manual.^14^ If a display is used for the primary mammographic interpretation from units across multiple facilities, it must comply with the requirements of each applicable manual.^15^

## 5 SPECIAL CONSIDERATIONS FOR REMOTE RWS

Mammography facilities and personnel are responsible for ensuring quality in the production, processing, and interpretation of mammograms. This requires that an RWS is compatible with all other equipment in the imaging chain. Remote RWS presents unique challenges to performing QM. The clinical task being performed on these devices is no different than in a traditional mammography reading room, but the lack of control over the reading environment, in combination with the remote setting, makes a properly planned and executed QM program even more critical.

a. Environmental conditions

Special considerations apply to the remote reading environment. While on‐site reading rooms are designed to meet specific viewing conditions, remote work settings are more variable. Many remote RWS are used in areas with windows or other light sources and may be in rooms used for other purposes, such as a dining or living room. The image interpreter's ability to detect small changes in luminance, especially at lower levels, may be reduced in the presence of excessive ambient light.^9^

The medical physicist should provide recommendations for creating a reading environment with appropriate ambient light levels. The medical physicist should also be involved in the evaluation of light sources, paying particular attention to sources of specular reflection. This includes encouraging the elimination of discrete light sources directly incident on a display surface, as well as the reduction of illumination provided by diffuse light sources to appropriate levels. This may include covering windows or turning off certain lights.

Evaluation of ambient lighting conditions should include both quantitative and qualitative assessments. While some of these evaluations may be performed automatically (e.g., a built‐in display illuminance meter), the medical physicist, or person under the general supervision of the medical physicist, should be responsible for in‐person evaluation of the ambient conditions.

b. Tests requiring user interaction

There are QA activities and QC evaluations that cannot be performed remotely. Certain qualitative tests require a user to evaluate test patterns, and some quantitative tests require the use of external measurement equipment. For example, testing internal photometers by comparing their measurements with those from a calibrated, external device is essential for validating the accuracy of internal photometer output, as well as maintaining compliance for systems with automated calibration.

As with the evaluation of environmental conditions, the medical physicist should be responsible for ensuring that in‐person tests are conducted appropriately, either by performing them personally or by delegating this work to a trained individual operating under the medical physicist's supervision.

## 6 RECOMMENDED SUPPORT, TRAINING, AND OVERSIGHT

a. IT support

Support from information technology (IT) staff is critical for the successful implementation of an RWS display QM program. Many displays and workstations support the use of manufacturer, vendor, or third‐party software to facilitate data collection and test pattern display, but the medical physicist's ability to install or modify such software, troubleshoot connectivity issues between the RWS and online QM tools, or correct issues with the workstation itself is likely limited by local IT security restrictions. The medical physicist should engage with their local IT group to create a partnership that will improve the implementation of a QM program. Such a partnership will also help facilitate communication between the IT group and the medical physicist and help alert the physicist to changes that require follow‐up testing.

b. Recommended training for QA/QC

The medical physicist may train other individuals to qualitatively analyze visual test patterns, collect quantitative data, and assess appropriate lighting conditions. The recommended level of oversight for QA/QC procedures is commensurate with the complexity of the task, the likelihood of failure, and the clinical impact of such a failure. The medical physicist holds ultimate responsibility for the development and management of an effective QM program. Training that will most benefit the QM program includes:

1. interpretation of visual test patterns,
2. collection of quantitative luminance measurements, and
3. evaluation of ambient light conditions.

Qualitative QC tests involve visually assessing test patterns. These can be performed quickly and allow for more frequent assessment of display performance between more comprehensive quantitative tests. The medical physicist should establish a training program for all individuals tasked with reviewing test patterns. At a minimum, the individual must demonstrate competency with all relevant pattern features, including those that may only rarely fail. Although the individuals performing these tests are not required to have the same level of knowledge as the medical physicist, they should always be able to identify a failure for each test pattern feature. While multiple patterns may be appropriate for some tests, the medical physicist should establish a single pattern for each test and train the individuals with example modes of failure. Training documentation and reference material are both recommended so the individuals performing the tests can feel confident in their task and to ensure that all users are performing the tests consistently.

Quantitative luminance measurements may be performed with either automated software or manually with an external photometer. In either case, the medical physicist must confirm the training of anyone performing these tasks. Similar to visual test patterns, the individuals should be able to identify data that suggest the display performance is compromised. The individuals collecting luminance data should understand when to contact the medical physicist for guidance during a test and be able to identify common failures.

The final area of recommended training is in the ability for individuals to properly assess ambient lighting conditions. This may be via the use of a visual test pattern (e.g., TG18‐AD), software with a forced choice selection, or other visual tools. The integrated illuminance meter in some displays that informs the reader when room lighting conditions exceed an acceptable threshold may not be sufficient for evaluating overall lighting conditions and specular reflection. A QM program should establish a single method for use at all workstations, allowing consistency and efficiency in training for the individuals performing the tests.

c. Recommended training for interpreting physicians

As the end users of RWS display, interpreting physicians is a critical part of the image review chain. Accordingly, they should be aware of general best practices in technology performance and environmental conditions when interpreting images. The medical physicist should work with their physician partners to establish training documentation and practices so interpreting physicians can participate in the general QM of the RWS displays. Most importantly, the interpreting physicians should be aware of the rationale for ambient lighting recommendations. They should also be able to recognize and communicate potential issues to the medical physicist. In the case of a remote reading environment, the interpreting physicians may also participate in the collection of QC test data so long as they've undergone similar training under the supervision of the medical physicist, just as with any other individual tasked with this work. This includes the tasks listed above in Section 6.b.

Ultimately, the interpreting physicians must share responsibility for an effective RWS display QM program. While the medical physicist may design and administer a QM program, the interpreting physician is the day‐to‐day user of the RWS display. Whether it is reporting on possible issues before they are discovered at testing, or correctly maintaining environmental conditions in a remote setting, the interpreting physicians are critical to the effectiveness and success of a QM program.

## 7 RECOMMENDED QA AND QC PROCEDURES

Table 1 outlines the recommended routine QA and QC procedures for mammography RWS displays, their frequencies, and the level of supervision that a medical physicist should provide. The tests, frequencies, and oversight levels listed in this document are not intended to supplant existing requirements. If a display or RWS is repaired, any tests relevant to the performance that may be affected by the repair should be performed. The tests relevant to a specific repair may vary by display. For example, many modern displays store the grayscale lookup table (LUT) on the display itself rather than the workstation. For such displays, the medical physicist may determine it is appropriate to omit certain tests when the workstation computer (and not the display) is replaced. The medical physicists should be familiar with the equipment they support so they can identify tests relevant to equipment changes or repairs. If a display or RWS is replaced, the medical physicist must determine what testing is necessary prior to use for clinical image interpretation.

**TABLE 1:** Recommended QC and QA procedures for mammography RWS displays.

Minimum recommended frequency	Medical physicist supervision^a^	QC/QA procedure(s)	Quality metric(s) evaluated	Test Pattern(s)/equipment
Quarterly	General	Qualitative ambient luminance/illuminance	Ambient lighting conditions	TG18‐AD TG270‐sQC TG270‐pQC TG18‐OIQ
		Qualitative luminance response, display resolution	Low‐contrast performance, bit depth, pixel mapping	TG270‐sQC TG270‐pQC TG18‐OIQ
		Qualitative uniformity	Visibility of clinically‐relevant non‐uniformities	TG270‐ULN TG18‐UN
Annually and acceptance	General	Quantitative luminance response, min/max luminance, color accuracy/tracking^b^	Luminance calibration accuracy, stability	Internal photometer, or external photometer and TG270‐ULN TG18‐LN TG270‐sQC^c^ TG270‐pQC^c^
		Quantitative ambient illuminance and/or luminance^d^	Ambient lighting conditions	Illuminance meter or telescopic photometer
		Internal photometer correlation (if applicable)	Accuracy of internal display photometer	External photometer, vendor software
	Personal	Meeting with LIP (virtual or in‐person)	Discussion of QM program	NA
		Review of display QM program	Medical physicist review of QA and QC data	NA
Acceptance and troubleshooting	General	Quantitative uniformity	Luminance uniformity	Photometer, TG270‐ULN TG18‐UN
		Mammography QC phantom evaluation	PACS, mammography unit, RWS connectivity	Clinical QC phantom image from one mammography unit
Vendor‐automated tests/calibration^e^	Variable	Variable	Variable	Variable

Abbreviations: LIP, lead interpreting physician; PACS, picture archiving and communication system; QA, quality assurance; QC, quality control; QM, quality management; RWS, review workstations.

^a^ Supervision definitions follow AAPM AP 125‐A. In all cases, the medical physicist must review instances of failures for the determination of the next steps. Under general supervision, the medical physicist must periodically review all results.

^b^ Color accuracy/tracking data may be collected simultaneously with luminance data if the photometer used also records color information. If this information is not available simultaneously, color accuracy/tracking may be performed at acceptance and for troubleshooting only.

^c^ Use requires the ability to zoom/pan the test pattern.

^d^ At least one of either ambient luminance or illuminance should be measured.

^e^ Vendor‐automated tests/calibrations vary by vendor. In some cases, the automated tests may be equivalent to other tests being performed manually in this table. The medical physicist should determine which tests are equivalent and if some may be replaced. The medical physicist should take care to evaluate the automated tests for equivalence prior to replacing manual tests. In the case where two equivalent tests are prescribed at different frequencies, the higher frequency should be used.

Details about many of the tests listed in Table 1 are available in AAPM Task Group Report 270^9^. Appropriate performance criteria should be based on recommendations from the display manufacturer or relevant accreditation organizations. Performance criteria not specified by the manufacturer or accreditation organizations should be based on national or professional guidelines and documented by the medical physicist.

The QM program for a facility should include an annual meeting between the medical physicist and the lead interpreting physician (LIP) for that facility. The purpose of this meeting is to provide an opportunity for the medical physicist and LIP to discuss any upcoming changes in equipment, including equipment location, personnel, and/or any operational concerns. This is not meant to be a review of specific QC data; rather, it is meant to establish and maintain adequate communication between the medical physicist and the LIP. This meeting may take place in person or virtually, either by telephone or video conference. Other individuals (e.g., lead technologist, administrator, IT support) vital to the QM program should be included at the discretion of the medical physicist or LIP. The date, time, and meeting participants should be documented.

The medical physicist is responsible for periodic review of the data collected as part of the procedures outlined in Table 1, regardless of whether they are personally collecting those data or providing supervision to the individual collecting data. A review by the medical physicist does not necessarily have to occur immediately after a test is performed. Any individual collecting data should be sufficiently trained so that they can alert the medical physicist to any test failures. Any corrective actions and timeframes following the failure of a test must be specified as part of the QM program. The purpose of the medical physicist review is to evaluate the overall QM program and identify data trends that may indicate upcoming performance issues. While a medical physicist may choose to review results more often, this review should occur at least annually. Such a review may be part of a comprehensive review of a facility's entire QM program, as required under MQSA.^16^

## AUTHOR CONTRIBUTIONS

This guideline was developed by the Medical Physics Practice Guideline Working Group 17.a of the Professional Council of the AAPM. Each author participated in conversations to develop the recommendations contained within this report. They also reviewed the manuscript at multiple steps of the writing process, including after review by AAPM membership.

## CONFLICT OF INTEREST STATEMENT

The Chair of the Medical Physics Practice Guideline Working Group 17.a has reviewed the Conflict‐of‐Interest statement on file for each member of Medical Physics Practice Guideline Working Group 17.a and determined that disclosure of potential Conflicts of Interest is an adequate management plan. The members of Medical Physics Practice Guideline Working Group 17.a listed above attest that they have no potential Conflicts of Interest related to the subject matter or materials presented in this document.
